# Management of chronic anal fissure: results of a national survey among gastrointestinal surgeons in the Netherlands

**DOI:** 10.1007/s00384-022-04115-9

**Published:** 2022-02-22

**Authors:** Danielle A. van Reijn-Baggen, Lisette Dekker, Henk W. Elzevier, Rob C. M. Pelger, Ingrid J. M. Han-Geurts

**Affiliations:** 1 Department of Surgery, Proctos Clinic, Bilthoven, The Netherlands; 2 Department of Urology, Leiden University Medical Center, Leiden, The Netherlands; 3 Department of Surgery, Amsterdam University Medical Center, Location AMC, Amsterdam, The Netherlands; 4 Department of Medical Decision Making, Leiden University Medical Center, Leiden, The Netherlands

**Keywords:** Chronic anal fissure, Pelvic floor, Treatment, Surgery, Botulinum toxin

## Abstract

**Background:**

Chronic anal fissure (CAF) is a common, bothersome condition frequently accompanied by pelvic floor complaints. Despite current guidelines, optimal management is challenging. The aim of this study is to evaluate current management of CAF among gastrointestinal surgeons in the Netherlands.

**Methods:**

Dutch gastrointestinal surgeons and residents were sent a survey invitation by email, which was available online between June 2021 and September 2021. The questionnaire consisted of 21 questions concerning work experience, physical examination, diagnostic and surgical techniques, and follow-up.

**Results:**

Overall, 106 (33%) respondents completed the survey. Most respondents (59%) had at least 10 years of experience in treating CAF. Only 23% always addressed pelvic floor complaints. Fifty-one percent performed digital rectal examination and 22% always, or almost always, examined the pelvic floor muscles. Most respondents started treatment with fibers and/or laxatives and ointment (96%). Diltiazem was in 90% the preferred ointment. Twenty-two percent referred patients for pelvic floor physical therapy. Botulinum toxin was in 54% performed under general or spinal anesthesia or sedation. The surgical procedure of choice was fissurectomy (71%) followed by lateral internal sphincterotomy (27%). Fissurectomy was in 51% always combined with botulinum toxin. Fifty-seven percent of the respondents preferred a physical follow-up appointment.

**Conclusion:**

Guideline recommendations are largely followed in the Netherlands, starting with conservative measures followed by surgical procedures. Surgeons do not consistently assess pelvic floor complaints, nor do they routinely examine the pelvic floor muscles. Awareness of pelvic floor dysfunctions is important to refer patients for pelvic floor physical therapy.

## Introduction

Chronic anal fissure (CAF) is defined as a longitudinal ulcer in the squamous epithelium with persisting symptoms for longer than 4 to 6 weeks or recurrent fissures [1]. Patients usually experience anal pain, during and immediately after defecation, which may last several hours and therefore has a substantial impact on daily activities and quality of life [2]. Despite current Dutch and international guidelines, optimal management of CAF is quite challenging, mainly because of its recurrent nature, therapy compliance, and the variety of non-operative and operative treatments [3, 4].

Initial conservative management comprised lifestyle advice, fiber intake, and/or use of laxatives and ointments. The use of ointments is aimed at reducing elevated internal sphincter tone. Botulinum toxin (BT) can be considered as an alternative or as a step-up approach when standard conservative therapy fails [3, 4]. In addition, various surgical procedures are possible such as fissurectomy, advancement flap repair, and lateral internal sphincterotomy (LIS). Currently, LIS is considered the golden standard [1, 4].

Although most anal fissures heal spontaneously or with conservative measures, a percentage tends to recur or persist. A proportion of these patients have a history of constipation and obstructed defecation due to an unrecognized pelvic floor dysfunction such as dyssynergia [5]. Pelvic floor dysfunctions are associated with urological, bowel, gynecological and sexual complaints and chronic pelvic pain and can be treated with pelvic floor physical therapy [6]. It is unknown if surgeons treating these patients are sufficiently aware of this condition in patients with CAF.

Although Dutch and international guidelines are largely based on high-quality evidence, recommendations are ambiguous. As a result, there is variation in clinical practice. The aim of this study is to evaluate current practice in the management of CAF among gastrointestinal surgeons in the Netherlands.

## Materials and methods

This study was performed and reported according to the Checklist for Reporting Results of Internet E-Surveys (CHERRIES) [7]. As this study did not apply the Medical Research Involving Human Subjects Act (WMO), approval by the ethics committee was not required.

The survey was written in Dutch, consisted of 21 questions and was created using a web-based program called Survio (www.survio.com). The survey was tested for completeness, usability, and technical functionality before submission. The closed-survey was sent by email to all members of the Dutch Working Group Coloproctology as well as to gastrointestinal surgeons, fellows, and residents of each hospital in the Netherlands. We used the email database of our previous survey among Dutch gastrointestinal surgeons [8]. The survey was accompanied by an invitation email explaining the objectives of the study and length of time of the survey (< 10 min). One reminder email was sent after 4 days, the second after 10 weeks. The survey was available online from June 25th, 2021, to September 30th, 2021.

Survio automatically collected all the data after which the data were exported to a Microsoft Excel spreadsheet and then imported to SPSS (version 26.0). To prevent missing data, all questions were mandatory with automated skip logic. Data were analyzed using descriptive statistics.

## Results

In total, 329 invitations were sent by email and hundred-and-six (33%) surveys returned and were completely answered. The results of the survey are shown in Table 1.

**Table 1:** Results

**Respondents’ characteristics**	N (%)
What is your medical specialty?	
Gastrointestinal surgeon General surgeon Fellow Resident in training Physician assistant/nurse practitioner	86 (81) 7 (7) 2 (2) 8 (7) 3 (3)
What type of hospital are you working?	
*Academic* *Non-academic (peripheral)* *(Private) clinic*	4 (4) 94 (89) 8 (7)
How many years of work experience do you have as a medical specialist in the treatment of CAF?	
*1–5 years* *5–10 years* *10–20 years* *> 20 years*	19 (18) 24 (23) 35 (33) 28 (26)
How many procedures for CAF (incl botulinum toxin) do you perform per year?	
*0–10* *10–30* *30–50* *> 50*	41 (39) 41 (39) 19 (18) 5 (5)
**Medical history and physical examination**	
How often do you ask a patient with CAF about pelvic floor complaints (gynaecology, urology, sexuology)? *SC?	
*Never/almost never* *In less than half of the cases* *In more than half of the cases* *Almost always/always*	30 (28) 38 (36) 14 (13) 24 (23)
In case you expect CAF by medical history, which physical examination and/or diagnostics do you do? *MC	
*None* *Inspection* *Digital rectal examination* *Proctoscopy* *Endo-anal ultrasound*	1 (1) 103 (97) 54 (51) 24 (23) 6 (6)
Do you examine the pelvic floor muscles by a patient with CAF (squeeze, relaxation and push of the levator ani muscle and external anal sphincter)? *SC	
*Never/almost never* *In less than half of the cases* *In more than half of the cases* *Almost always/always*	39 (37) 26 (24) 18 (17) 23 (22)
**Treatment**	
Which treatment do you initiate when treating a patient with CAF? (assuming the general practitioner has not already done this) *MC	
*Lifestyle advice by nutrition advice and toilet behaviour* *Fibers/laxatives and ointment* *Pain medication (local and/or systemic)* *Pelvic floor physical therapy* *Botulinum toxin*	79 (74) 102 (96) 43 (41) 23 (22) 2 (2)
Which ointment do you prescribe for CAF? *SC	
*Lidocaine* *Isosorbide dinitrate* *Diltiazem* *Other*	1 (1) 9 (8) 96 (90) 0 (0)
In case of isosorbide dinitrate or diltiazem, what was your recommendation concerning duration of application? (number)	
*16 weeks* *12 weeks* *8 weeks* *6 weeks* *4 weeks* *3 weeks* *2 weeks*	1 (1) 29 (27) 13 (12) 59 (56) 1 (1) 1 (1) 2 (2)
Do you feel you have enough time to instruct and advice the patient regarding the use of laxatives, lifestyle and ointment? *SC	
*Never/almost never* *In less than half of the cases* *In more than half of the cases* *Almost always/always*	4 (4) 7 (7) 19 (18) 76 (72)
How do you perform the botulinum toxin (BT) injections? *SC	
*Outpatient clinic, without anesthesia* *Outpatient clinic, with local anesthesia* *General- or spinal anesthesia or sedation* *Not applicable, I do not perform this procedure*	34 (32) 4 (4) 45 (42) 23 (22)
How often do you repeat BT injections? *SC	
*One time* *Two times* *More than two times* *I do not repeat*	16 (19) 41 (49) 22 (27) 4 (5)
Do you simultaneously give BT in the levator ani muscle when treating CAF? *SC	
*Never/almost never* *In less than half of the cases* *In more than half of the cases* *Almost always/always*	63 (76) 13 (16) 6 (7) 1 (1)
What is your preferred surgical procedure for CAF (except BT)? *SC	
*Fissurectomy* *Lateral internal sphincterotomy (LIS)* *Advancement flap repair*	59 (71) 22 (27) 2 (2)
In case you perform a fissurectomy, do you simultaneously give BT intersphincteric? *SC	
*Never/almost never* *In less than half of the cases* *In more than half of the cases* *Almost always/always*	15 (18) 7 (8) 19 (23) 42 (51)
In case you perform BT under anesthesia, do you simultaneously perform a fissurectomy? *SC	
*Never/almost never* *In less than half of the cases* *In more than half of the cases* *Almost always/always*	24 (29) 15 (18) 22 (27) 22 (27)
**Follow-up**	
How do you manage the follow-up after starting a treatment? *SC	
*No follow-up* *Physical appointment* *Telephone call* *According to the needs of the patient*	0 (0) 60 (57) 22 (21) 24 (23)
How many times did you refer a patient with CAF to another specialist last year? (number)	
*0 times* * 1–5 times* * 6–10 times*	61 (58) 42 (40) 3 (3)
What percentage of your patients do you estimate to be symptom-free a year after starting the treatment? *SC	
* 0–25%* *25–50%* *50–75%* *75–100%* *I do not know*	0 (0) 9 (8) 60 (57) 33 (31) 4 (4)
Do you feel you can treat patients with CAF satisfactorily? *SC	
*Never/almost never* *In less than half of the cases* *In more than half of the cases* *Almost always/always*	0 (0) 2 (2) 72 (68) 32 (30)

*CAF* chronic anal fissure, *BT* botulinum toxin, *SC* single choice, *MC* multiple choice

Eighty-one percent of the respondents were gastrointestinal surgeons, and 89% worked in a general hospital. Fifty-nine percent had at least 10 years of experience with treating CAF, and 61% performed more than 10 procedures for CAF per year, including botulinum toxin (BT).

From the respondents, 28% never or almost never asked, and only 23% always or almost always asked for complaints in other domains of the pelvic floor. Half of the respondents performed digital rectal examination, and 23% performed proctoscopy. Only 22% of the respondents indicated that they always, or almost always, performed physical examination of the pelvic floor muscles.

Ninety-six percent started treatment with fibers and/or laxatives and ointment. In 90% of the respondents, diltiazem was the preferred ointment. Fifty-six percent prescribed ointment for a period of 6 weeks. Most of the respondents (72%) felt they had enough time to give the patient instructions or advice regarding the use of laxatives, lifestyle, and ointment. Twenty-two percent of the respondents referred to a pelvic floor physical therapist, and they always combined this with fibers and/or laxatives.

BT injections were given by 77% of the respondents mainly under general or spinal anesthesia or sedation (42%). Almost half of the respondents repeated BT injections twice, and more than 76% never performed BT in the levator ani muscle.

Fissurectomy was the most popular operative procedure (71%), followed by LIS (27%). More than half of the respondents always, or almost always, used BT intersphincteric in case they performed a fissurectomy.

Fifty-seven percent scheduled a physical follow-up check in the outpatient clinic. A percentage of 57% estimated their patients to be symptom-free after 1 year in 50–75% of the cases.

## Discussion

Implementation of Dutch and international guidelines for chronic anal fissure in daily practice varies. The present study provides an overview of the current approach in the management of CAF among gastrointestinal surgeons in the Netherlands.

The pelvic floor plays a major role in defecation and continence. Furthermore, pelvic floor dysfunctions are prevalent in patients with chronic anal pain syndromes [9]. However, 28% of the respondents never or almost never asked for any pelvic floor complaints in the patients with CAF. We feel that knowledge about pelvic floor dysfunctions is beneficial in the treatment of anorectal disorders since this might result in a referral to another specialist in an early stage.

Digital rectal examination was performed by half of the respondents, and 37% never or almost never examined the pelvic floor muscles. In case of expecting a CAF, the reason for not performing digital rectal examination could be the assumption that it contradicted or should be kept to a minimum because of associated pain. However, careful digital rectal examination is important to obtain information on anorectal anatomy and function [5]. When identifying pelvic floor muscle dysfunction, patients can appropriately be referred to a pelvic floor physical therapist.

Most of the respondents is accustomed to start with conservative measures which is according to current guidelines [3, 4]. Most respondents did have enough time to give instructions in the consulting room. This is important, since information about patients’ complaints, lifestyle advice, laxative or ointment and its use require an explanation by the clinician [10].

Pelvic floor dysfunctions can effectively be treated with pelvic floor physical therapy, but only 22% of the respondents referred to this treatment modality, a missed opportunity. The clinical effect of pelvic floor physical therapy in patients with CAF is currently investigated by the Pelvic floor Anal Fissure (PAF) study.

More than half of the respondents (54%) performed BT injections under general or spinal anesthesia or sedation which is in accordance with a recent survey among members of the American Society of Colon and Rectal Surgeons (ASCRS) [11]. In current literature, there is no consensus on dose, site, or number of injections [12]. This corresponds with the results of our study showing no consensus on how often one should repeat BT.

In case BT was performed under anesthesia, only 27% always or almost always simultaneously performed fissurectomy, and another 27% does this in more than half of the cases. This is also comparable to the results of the survey among members of the ASCRS [11].

LIS is the preferred treatment for refractory anal fissures and is still considered the golden standard since LIS has superior healing rates [3, 4]. In our study, fissurectomy was the surgical procedure of choice in 71% of the respondents, followed by LIS (27%). Guideline recommendations differ on this subject. The ASCRS guideline favors LIS [4], the Dutch guideline, however, recommends LIS only for refractory fissures when previous treatment fails [3].

The follow-up was diverse in our survey. Twenty-one percent of the respondents stated that they scheduled a telephone call follow-up. This is quite interesting given the fact that CAF concerns a chronic disorder which is often accompanied by pelvic floor dysfunctions. A physical diagnostic follow-up will probably better monitor patients’ wellbeing and subsequently ensure that the patient does not end up in a vicious circle of pain again.

This study has some limitations that should be mentioned. First, the response rate of 33% may have caused non-response bias. Second, the questionnaire was sent to all the members of the Dutch Coloproctology Working group that consists of members that have large experience and affiliation in treating anorectal diseases. Of all respondents, 33% came from this group. This may have caused selection bias. Third, we used a non-validated questionnaire, and the respondents were self-reported. Self-reports may have resulted in an overestimation of history-taken practices and to our knowledge, validated questionnaires are not available in this field.

## Conclusion

Guideline recommendations in treating CAF are largely followed and consistent among most gastrointestinal surgeons in the Netherlands. Initial treatment consists of conservative measures followed by surgical procedures. Surgeons do not consistently assess pelvic floor complaints, nor do they routinely examine the pelvic floor muscles. Awareness of pelvic floor dysfunctions in patients with CAF is important to refer patients for pelvic floor physical therapy.

## Data Availability

The dataset used during the study is available upon reasonable request from the corresponding author.
